# Value of MRI-based semi-quantitative structural neuroimaging in predicting the prognosis of patients with idiopathic normal pressure hydrocephalus after shunt surgery

**DOI:** 10.1007/s00330-022-08733-3

**Published:** 2022-04-30

**Authors:** Jiakuan Chen, Wenjie He, Xiejun Zhang, Minrui Lv, Xi Zhou, Xiaolin Yang, Haihua Wei, Haiqin Ma, Hongbing Li, Jun Xia

**Affiliations:** 1 Department of Radiology, The First Affiliated Hospital of Shenzhen University, Shenzhen University, Shenzhen Second People’s Hospital, 3002 SunGang Road West, Shenzhen, 518035 Guangdong Province China; 2 Guangzhou Medical University, Guangzhou, China; 3 Department of Neurosurgery, The First Affiliated Hospital of Shenzhen University, Shenzhen University, Shenzhen Second People’s Hospital, 3002 SunGang Road West, Shenzhen, 518035 Guangdong Province China; 4 Guangdong Medical University, Zhanjiang, China; 5 Shantou University Medical College, Shantou, China; 6 Department of Radiology, Fuyong People’s Hospital, Baoan District, Shenzhen, 518103 Guangdong Province China

**Keywords:** Idiopathic normal pressure hydrocephalus, DESH score, iNPH Radscale, Structural neuroimaging

## Abstract

**Objectives:**

To explore the value of structural neuroimaging in predicting the prognosis of shunt surgery for idiopathic normal-pressure hydrocephalus (iNPH) using two different standard semi-quantitative imaging scales.

**Methods:**

A total of 47 patients with iNPH who underwent shunt surgery at our hospital between 2018 and 2020 were included in this study. The modified Rankin Scale (mRS) and iNPH grading scale (iNPHGS) were used to evaluate and quantify the clinical symptoms before and after shunt surgery. The disproportionately enlarged subarachnoid space hydrocephalus (DESH) and iNPH Radscale scores were used to evaluate the preoperative MR images. The primary endpoint was improvement in the mRS score a year after surgery, and the secondary endpoint was the iNPHGS after 1 year. The preoperative imaging features of the improved and non-improved groups were compared.

**Results:**

The rates of the primary and secondary outcomes were 59.6% and 61.7%, respectively, 1 year after surgery. There were no significant differences in preoperative DESH score, iNPH Radscale, Evans’ index (EI), or callosal angle (CA) between the improved and non-improved groups. Significant correlations were observed between the severity of gait disorder and EI and the CA.

**Conclusions:**

The value of structural neuroimaging in predicting the prognosis of shunt surgery is limited, and screening for shunt surgery candidates should not rely only on preoperative imaging findings.

**Key Points:**

*• Early shunt surgery can significantly improve the clinical symptoms and prognosis of patients with idiopathic normal-pressure hydrocephalus (iNPH).*

*• Structural imaging findings have limited predictiveness for the prognosis of patients with iNPH after shunt surgery.*

*• Patients should not be selected for shunt surgery based on only structural imaging findings.*

**Supplementary Information:**

The online version contains supplementary material available at 10.1007/s00330-022-08733-3.

## Introduction

Idiopathic normal-pressure hydrocephalus (iNPH) is a senile syndrome of unknown etiology characterized by gait disorders, cognitive impairment, and urinary incontinence [1, 2]. Its incidence and disability rate increase significantly with age. On brain imaging, the main manifestation is the enlargement of the lateral ventricle [3], with normal cerebrospinal fluid (CSF) pressure. Early shunt surgery can significantly improve the clinical symptoms and patient prognosis [4]. At present, CSF tap test is still the main diagnostic method and for judging prognosis after iNPH surgery. However, it is an invasive test with complications such as low back pain, low intracranial pressure, headache, and low negative predictive value [5]. Patients with a negative CSF tap test may also benefit from surgery. Therefore, the study of non-invasive prognostic indicators for iNPH surgery has clinical significance.

Several scholars have conducted extensive research on the structural imaging features of iNPH. For example, the Japanese guidelines emphasize that the presence of disproportionately enlarged subarachnoid space hydrocephalus (DESH) in neuroimaging is an important indicator for the diagnosis of iNPH [6] and that the DESH sign reportedly plays a positive role in predicting the prognosis of shunt surgery [7]. Based on this, Shinoda et al developed the DESH score based on iNPH’s MRI features and explored its value in prognostic predictions for iNPH [8]. In their study, the DESH score included five items: Evans’ index (EI), Sylvian fissures, tight high convexity, callosal angle (CA), and focal sulcal dilatation. In addition, Kockum et al developed the iNPH Radscale to explore the relationship between imaging features and clinical symptoms of iNPH. Based on CT image features, it included seven items: the EI, Sylvian fissures, tight high convexity, CA, focal sulcal dilatation, temporal horns, and periventricular hyperintensities (PVH) [9]. However, the accuracy and practicability of traditional structural imaging in the diagnosis and prognosis of iNPH are still debated [10–13].

We hypothesized that there were differences in structural imaging between iNPH patients who improved and those who did not improve after shunt surgery. By comparing the difference of preoperative DESH score and iNPH Radscale score between the improvement and non-improvement groups, the value of structural imaging features in the prognostic evaluation of iNPH patients undergoing shunt surgery was clarified.

## Materials and methods

**Fig. 1:**
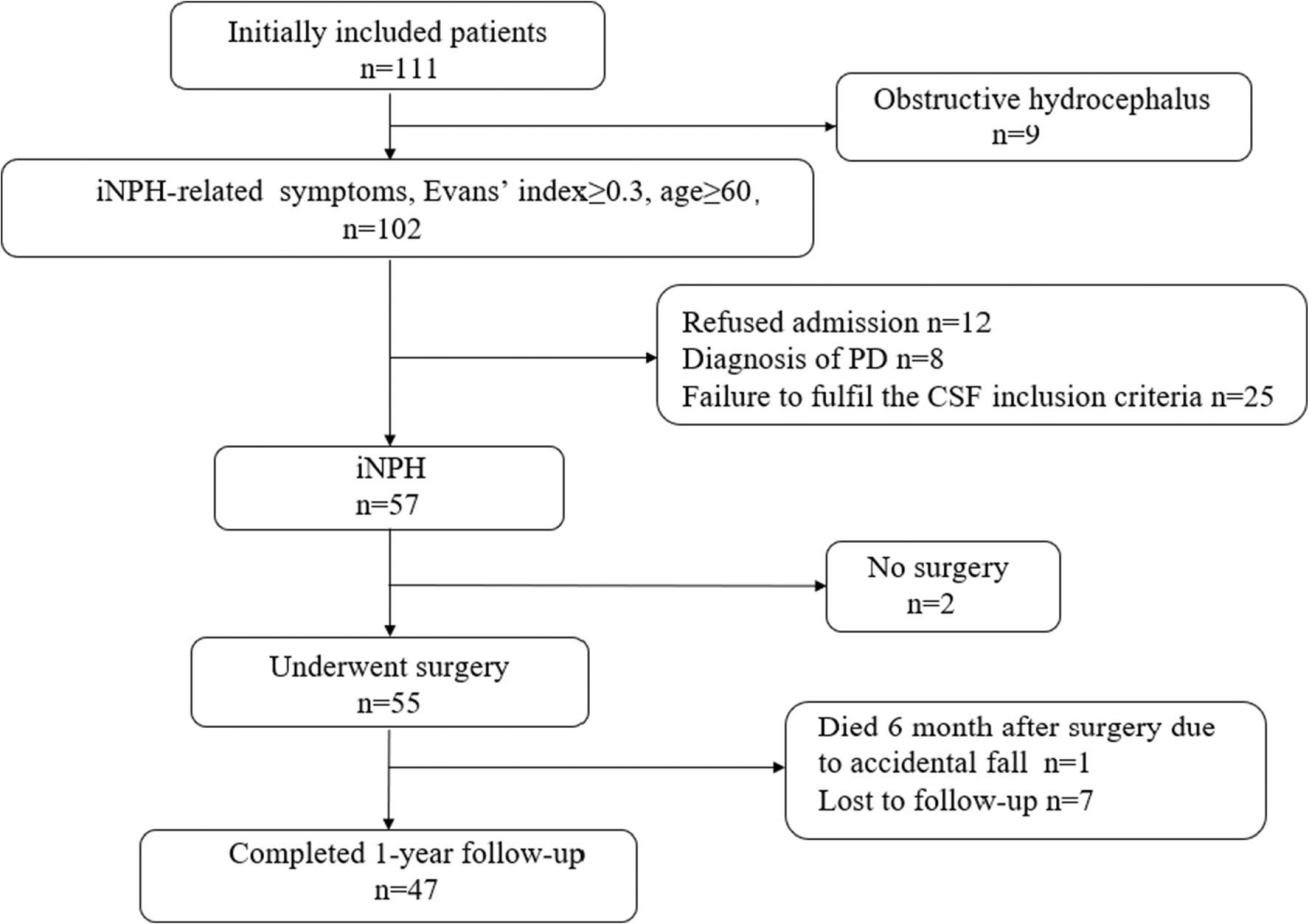
Flow chart for this study from the initial screening to the final analysis. iNPH, idiopathic normal-pressure hydrocephalus; PD, Parkinson’s disease; CSF, cerebrospinal fluid

The initial screening involved a clinical sample of 111 consecutive patients suspected of iNPH who underwent brain MRI examination at our hospital between January 2018 and December 2020. Figure 1 shows the flowchart for this study, from initial screening to the final analysis. This study initially included 111 patients who visited the hospital due to ≥ 1 of progressive gait disorder, cognitive impairment, and urinary incontinence, and who were evaluated by neurologists and underwent relevant head imaging examinations. Among them, nine patients were diagnosed with obstructive hydrocephalus after a detailed MRI examination. iNPH-related symptoms were confirmed in 111, and the imaging manifestations were signs of ventricular dilatation (EI ≥ 0.3). These patients were suspected of iNPH and admitted to the neurosurgery department for further examination and treatment; 12 refused admission due to family reasons. The hospitalized patients underwent more detailed examinations, including CSF biochemical and stress examinations and behavioral and cognitive examinations. Eight patients were diagnosed with Parkinson’s disease (PD) and were excluded, 25 patients were excluded because of failure to fulfill the CSF inclusion criteria, and 2 patients refused surgery; therefore, 55 patients underwent shunt surgery. During the 12 months of follow-up, one patient died after an accidental fall and seven were lost to follow-up because they moved to other hospitals in their hometown. Finally, the remaining 47 cases were evaluated. The protocol was approved by our hospital’s bioethics committee (approval no. KS20190114001).

For patient selection, we used the following criteria based on international standards [2] and actual conditions: (1) age ≥ 60 years; (2) ≥ 1 clinical manifestations of the triad of gait disorder, cognitive impairment, and urinary incontinence; (3) manifestations of ventricular enlargement (EI ≥ 0.3) on imaging, with the exclusion of other diseases that may cause it, such as traumatic brain injury, various types of cerebral hemorrhage, brain tumors, encephalitis, meningitis, and large-area cerebral infarction, among others; (4) CSF pressure ≤ 200 mmH_2_O on lumbar puncture, with normal CSF biochemical examination results; (5) positive response on the CSF tap test [14]; and (6) no severe cardiovascular and cerebrovascular diseases and other diseases that are contraindications for surgery. In addition, all patients underwent head MRI examination within 1 month before surgery, and a ventricular-abdominal shunt was selected for surgery.

### Clinical evaluation

All patients underwent comprehensive clinical examinations by a neurologist preoperatively and 12 months postoperatively according to standardized protocols [15]. To evaluate the outcomes of shunt surgery, we used the modified Rankin Scale (mRS) to assess the general level of disability and the overall situation [16]; the iNPH grading scale (iNPHGS) can be used to evaluate individual symptoms related to the triad of gait, cognition, and urination, and its total score represents the overall severity of clinical symptoms [17]. The primary outcome was an improvement in mRS ≥ 1 points (favorable outcome) a year after surgery, and the secondary outcome was an improvement in iNPHGS ≥ 1 point a year after surgery. We examined the effect of preoperative neuroimaging features on these two scores.

### MRI

All preoperative MRI scans were performed using a 3.0-T MRI scanner (Siemens Prisma) with a dedicated 20-channel head coil. The imaging protocol consisted of the following: (1) t1_mprage_sag_p2_iso sequence (3D-T1 weighted imaging)—TR/TE = 2300/3.55 ms; field of view = 240 × 240 mm, flip angle = 8°, slice thickness = 0.9 mm, slice gap = 13.4 mm, and scan time = 5 min 20 s; (2) transaxial T2 FLAIR sequence—TR/TE = 9000/81 ms; field of view = 220 × 220 mm; flip angle = 150°, slice thickness = 6.0 mm, slice gap = 0.7 mm, and scan time = 2 min 26 s. All measurements were performed digitally using a clinical picture archiving and communication system (PACS). Multiplanar reconstruction was performed interactively using the PACS for each image to obtain a coronal image. The preoperative images of all the participants were retrospectively evaluated by two experienced neuroradiologists who were blinded to clinical data. In case of inconsistent results, the image was re-evaluated until a consensus was reached.

**Fig. 2:**
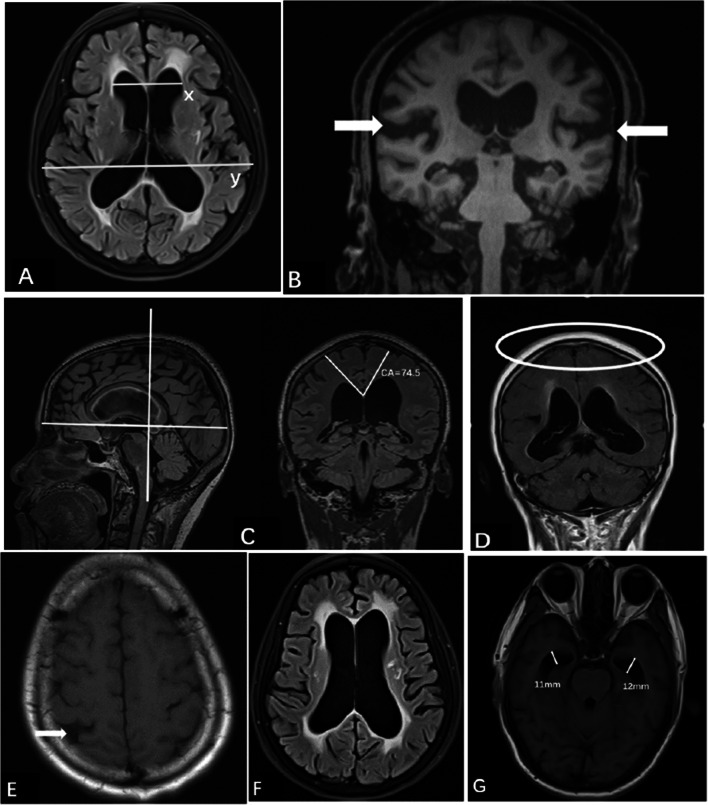
MR images of seven patients with iNPH. **A** Evans index = A/B. **B** Enlarged Sylvian fissures. Narrow medial sulci and two focally dilated sulci on the left side. **C** Callosal angle. **D** Tight high convexity. **E** Focally dilated sulci. **F** PVH graded as 2. **G** Dilated temporal horns. iNPH, idiopathic normal-pressure hydrocephalus; PVH, periventricular hyperintensities

In our study, neuroimaging features included the DESH score and the iNPH Radscale. The DESH score is based on five sub-items: EI, Sylvian fissures, tight high convexity, CA, and focal sulcal dilatation. Each item is assigned 0–2 points, with a total of 10 points. The iNPH Radscale assesses seven radiologic indexes, including EI, Sylvian fissures, tight high convexity, CA, focal sulcal dilatation, temporal horns, and periventricular hyperintensities. The scores range from 0 to 12. A comparison of the two is shown in Table 1. The image measurement methods are as follows (Fig. 2):

1. The EI was calculated as the ratio of the maximum diameter of the frontal horns of the lateral ventricles to the maximum inner diameter of the skull on the same plane of the transverse section (Fig. 2a) [18].
2. Sylvian fissure: the coronal images used for the Sylvian fissure ordinal were reconstructed at the level of the central part of the brain stem and angulated along the brain stem (Fig. 2b) [19].
3. The CA, the angle between the left and right corpus callosa and perpendicular to the anterior/posterior commissure plane, was measured on the coronal plane at the posterior commissure (Fig. 2c) [20].
4. Tight high convexity: the compression of the medial and/or high convexity cortex sulci (narrow sulci) was evaluated on coronal and transverse images (Fig. 2d) [21].
5. Focal sulcal dilatation: focal enlargement of the cortical sulci was visually evaluated on transverse sections (Fig. 2e) [22].
6. Periventricular hyperintensities (PVH): On T2-FLAIR images, PVH was graded as “not present,” “present around the frontal horns (as a cap),” or “diffusely extending around the lateral ventricles” based on quantitative evaluation with Fazekas’ score (Fig. 2f) [23].
7. Temporal horns: The maximum diameter of the temporal horns was measured in millimeters for each side on the transverse images, and the averages for the left and right were calculated (Fig. 2g) [19].

**Table 1:** Comparison of the DESH and iNPH Radscale scores

	DESH score	iNPH Radscale
Total	10	12
EI	0, < 0.3 1, 0.3–0.35 2, > 0.35	0, ≤ 0.25 1, > 0.25–0.3 2, > 0.3
Sylvian fissures	0, normal or wider than normal 1, slight dilatation or unilateral 2, bilateral dilatation	0, normal 1, enlarged
Narrow sulci	0, normal or wider than normal 1, slight compression 2, definitive compression	0, normal 1, parafalcine 2, vertex
CA	0, >100° 1, 90–100° 2, < 90°	0, > 90° 1, 90 to > 60° 2, ≤ 60°
Focally enlarged sulci	0, not present 1, some present 2, many present	0, not present 1, present
Temporal horns	NA	0, < 4 mm 1, 4 to < 6 mm 2, ≥ 6 mm
Periventricular hyperintensities	NA	0, not present 1, frontal horn caps 2, confluent areas

*DESH* disproportionately enlarged subarachnoid space hydrocephalus, *iNPH* idiopathic normal pressure hydrocephalus, *EI* Evan’s index, *CA* callosal angle

### Statistical analysis

All calculations were performed using SPSS version 26.0 (IBM), ICC, and κ inter-observer reliability. The Shapiro-Wilk test of normality was used to determine the data distribution. A *t*-test was used to identify significant differences among normally distributed data between the improvement and non-improvement groups, such as age. Similarly, the Mann-Whitney test was used to test the difference in non-normally distributed parameters, such as imaging parameters. The Wilcoxon signed-rank test was used to analyze the 1-year changes in clinical scores. Associations between outcome and MRI variables were assessed using logistic regression models, with results presented as odds ratios with 95% confidence intervals (CIs) in a forest plot. Receiver operating characteristic curves (ROCs) were used to evaluate the predictive effectiveness of the two scores on the results. The Spearman correlation coefficient was used to determine the correlation between the neuroimaging and clinical parameters. Statistical significance was set at 0.05 (two-tailed).

## Results

The demographic characteristics and preoperative imaging parameters of the 47 patients included in the study are shown in Table 2. Of the participants, 49% were men. The age at the time of the shunt surgery was 69.2 ± 5.9 years. The median (interquartile range, IQR) duration of the symptoms (having at least two or more of the triad) before imaging was 12.0 (6.0–36.0) months. Based on the iNPHGS, the typical preoperative symptoms were distributed as follows: 47 patients had gait disturbance, 45 had cognitive impairment, 37 had urinary symptoms, and 37 had the classic triad.

**Table 2:** Demographic characteristics and preoperative imaging parameters of the 47 patients with iNPH

Characteristic	Value
Demographics and characteristics	
Age (year)	69.2 ± 5.9
Sex, male (%)	23 (49%)
Symptom duration (median) (IQR) (month)	12.0 (6.0–36.0)
Medical history	
Hypertension	26 (56%)
Diabetes	11 (24%)
Prevalence of symptom	
Gait only	2
Cognitive only	0
Urinary only	0
Gait and urinary	0
Gait and cognitive	8
Cognitive and urinary	0
Triad (all 3 symptoms)	37
Preoperative MR imaging findings	
DESH score	6.0 (4.0–6.0)
iNPH Radscale	8.0 (6.5–10.0)
EI	0.36 (0.33–0.41)
CA	84.0 (75.0–95.5)
Temporal horn	7.4 (6.2–9.9)

*iNPH* idiopathic normal pressure hydrocephalus. Nonparametric data are given as median (interquartile range)

Table 3 shows the interrater reliability. For continuous variables, the reliability ranged between 0.95 and 0.98 (ICC), and for variables on an ordinal scale, between 0.56 and 0.73 (κ). Except for the focal sulcal dilatation, all evaluations showed substantial consistency. For all patients, the preoperative EI was > 0.3. The median DESH score and iNPH Radscale were 6.0 (4.0–6.0) and 8.0 (6.5–10.0), respectively. Severe PVHs (white matter hyperintensities extending from the paraventricular to the deep white matter) were observed in 48% of the patients. The callosal angle was < 90° in 64% of patients. A temporal horns’ diameter ≥ 6 mm was observed in 80% of the patients.

**Table 3:** Interrater reliability between two independent investigators for all imaging findings

Image feature	Reliability
Evans’ index (ICC)	0.98
Callosal angle (ICC)	0.95
Sylvian fissure (κ)	0.62
Tight high convexity (κ)	0.67
Focal sulcal dilation (κ)	0.56
PVH (κ)	0.73
Temporal horns (ICC)	0.98

*ICC* intraclass coefficient, *κ* weighted kappa, *PVH* periventricular hyperintensities

**Fig. 3:**
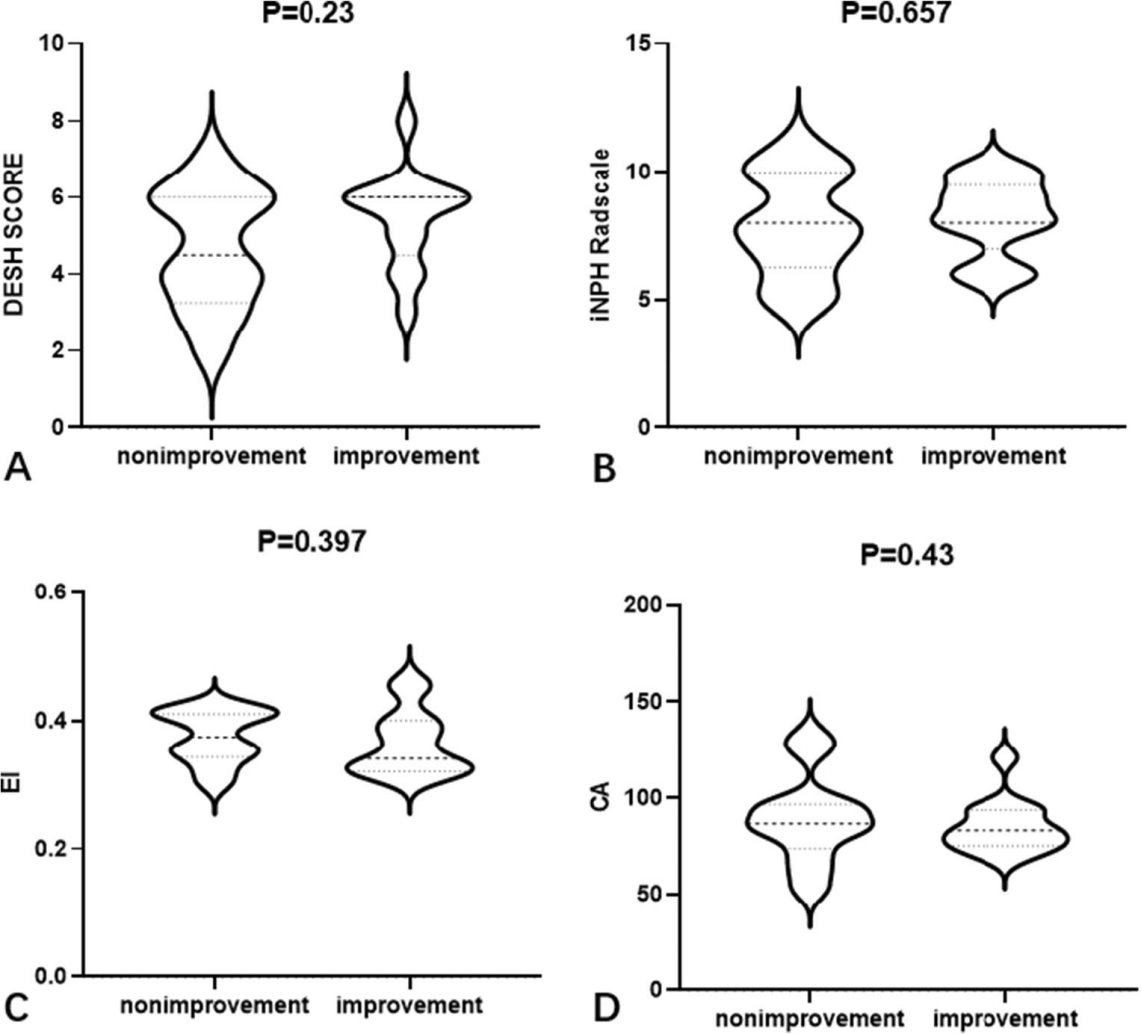
Differences in estimated MR imaging parameters between the non-improvement and improvement groups based on the mRS scores after one year. **A** DESH scores for the two groups. **B** iNPH Radscale scores for the two groups. **C** EIs for the two groups. **D** CAs for the two groups. EI, Evan’s index; CA, Callosal angle

An mRS improvement ≥ 1 within the first year after shunt placement was the primary index. iNPH patients were divided into an improvement group and a non-improvement group; 28 patients (59.6%) showed improvement, and the median (IQR) mRS improved from 2.0 (2.0–3.0) to 2.0 (1.0–3.0) (*p* = 0.039). The preoperative imaging parameters and clinical results showed that the median DESH scores of the improvement (Md = 6.0, IQR 4.5–6.0) and non-improvement (Md = 4.5, IQR 3.25–6.0) groups were not significantly different (*p* = 0.230; Table 4). Similarly, the improvement (Md = 8.0, IQR 7.0–9.5) and non-improvement (Md = 8.0, IQR 6.25–10.0) groups showed no significant differences in iNPH Radscale scores (*p* = 0.657), EI (*p* = 0.397), and CA (*p* = 0.43; Fig. 3).

**Table 4:** Preoperative characteristics of patients with and without an improved mRS score at 1 year after surgery

Characteristic	Improvement (*n* = 28)	No improvement (*n* = 19)	*p* value^†^
Demographic information			
Age (years)	68.3 ± 8.8	65.3 ± 9.4	0.413
Duration of symptoms (months)	12 (6.0–24.0)	13.5 (7–72)	0.476
Preoperative clinical outcomes			
mRS score	2.0 (2.0–3.5)	2.0 (1.0–3.0)	0.423
iNPHGS score	6.0 (5.0–7.5)	5.5 (3.3–7.0)	0.621
MRI findings			
DESH score	6.0 (4.5–6.0)	4.5 (3.25–6.0)	0.230
iNPH Radscale	8.0 (7.0–9.5)	8.0 (6.25–10.0)	0.657
EI	0.34 (0.32–0.40)	0.378(0.34–0.41)	0.397
CA	83.0 (75.0–93.5)	86.5 (73.5–96.8)	0.430

Nonparametric data are presented as median (interquartile range). Significant difference was determined using the Mann-Whitney *U* test

*iNPHGS* idiopathic normal pressure hydrocephalus grading scale, *MRI* magnetic resonance imaging, *mRS* modified Rankin Scale, *DESH* disproportionately enlarged subarachnoid space hydrocephalus, *EI* Evans’ index, *CA* callosal angle

The secondary outcome was an iNPHGS improvement ≥ 1 point. We found that 29 (61.7%) patients had improvement in the iNPHGS; the total iNPHGS improved from 6.0 (4.0–7.0) to 4.0 (2.5–7.0) (*p* = 0.011). Specifically, 24 patients showed improvement in gait, 20 showed improvement in cognitive ability, and 14 showed improvement in urination. Similarly, we divided the iNPH patients into improvement and non-improvement groups based on the iNPHGS scores (iNPHGS total, iNPHGS gait, iNPHGS cognitive, iNPHGS urinary) before and after shunt surgery, and the preoperative imaging parameters and clinical outcomes were compared. Similarly, there were no significant differences between patients with and without improvements in preoperative imaging parameters and clinical outcomes (Table 5).

**Table 5:** Preoperative characteristics of patients with and without improved secondary outcomes a year after surgery

Characteristic	iNPHGS total			iNPHGS gait			iNPHGS cognitive			iNPHGS urinary		
	Improvement (*n* = 29)	No improvement (*n* = 18)	*p* value	Improvement (*n* = 24)	No improvement (*n* = 23)	*p* value	Improvement (*n* = 20)	No improvement (*n* = 27)	*p* value	Improvement (*n* = 14)	No improvement (*n* = 33)	*p* value
Demographic information												
Age (years)	67.0 ± 8.8	66.8 ± 9.9	0.958	66.5 ± 9.5	67.2 ± 9.0	0.854	66.7 ± 8.9	67.1 ± 9.4	0.919	65.1 ± 8.1	67.6 ± 9.5	0.552
Duration of symptoms	12 (6–24)	12 (5–62)	0.807	12 (6–33)	12 (5–48)	0.765	12 (6–20)	15 (7–60)	0.301	6 (3–24)	12 (11–51)	0.357
Preoperative clinical outcomes												
mRS score	2.0 (2.0–4.0)	2.0 (1.8–3.0)	< 1.0	2.0 (1.0–3.3)	2.0 (2.0–3.0)	0.428	2.0 (2.0–4.0)	2.0 (1.3–3.0)	0.598	2.0 (1.0–4.0)	2.0 (2.0–3.0)	0.976
iNPHGS score	6.0 (5.0–8.0)	5.5 (3.0–7.0)	0.311	6.5 (4.8–7.3)	6.0 (3.0–7.0)	0.605	6.0 (5.0–8.5)	6.0 (3.3–7.0)	0.419	7.0 (6.0–8.0)	5.0 (3.0–7.0)	0.11
MRI findings												
DESH score	6.0 (5.0–6.0)	4.0 (3.0–6.3)	0.238	6.0 (4.0–6.3)	5.0 (4.0–6.0)	0.461	6.0 (4.5–6.0)	5.0 (3.3–6.0)	0.388	6.0 (5.0–6.0)	5.5 (4.0–6.0)	0.657
iNPH Radscale	8.0 (8.0–10.0)	7.5 (5.8–10.0)	0.397	8.0 (7.5–10)	8.0 (6.0–10.0)	0.643	8.0 (7.0–9.0)	8.0 (6.3–10.0)	0.803	9.0 (8.0–10.0)	8.0 (6.0–10.0)	0.244
EI	0.37 (0.33–0.41)	0.36 (0.32–0.41)	0.756	0.36 (0.34–0.41)	0.36 (0.32–0.41)	0.978	0.37 (0.34–0.43)	0.36 (0.32–0.41)	0.677	0.34 (0.32–0.45)	0.37 (0.33–0.40)	0.657
CA	83.0 (75.0–93.0)	91.0 (78.5–104.0)	0.177	83.5 (73.3–93.3)	86.0 (79.0–97.0)	0.285	84.0 (79.0–89.5)	85.5 (72.0–96.8)	0.677	84.0 (75.0–96.0)	85.0 (74.3–95.3)	0.79

Nonparametric data are presented as median (interquartile range). Significant difference was determined using the Mann-Whitney *U* test

*iNPHGS* idiopathic normal pressure hydrocephalus grading scale, *MRI* magnetic resonance imaging, *mRS* modified Rankin Scale, *DESH* disproportionately enlarged subarachnoid space hydrocephalus, *EI* Evans’ index, *CA* callosal angle

**Fig. 4:**
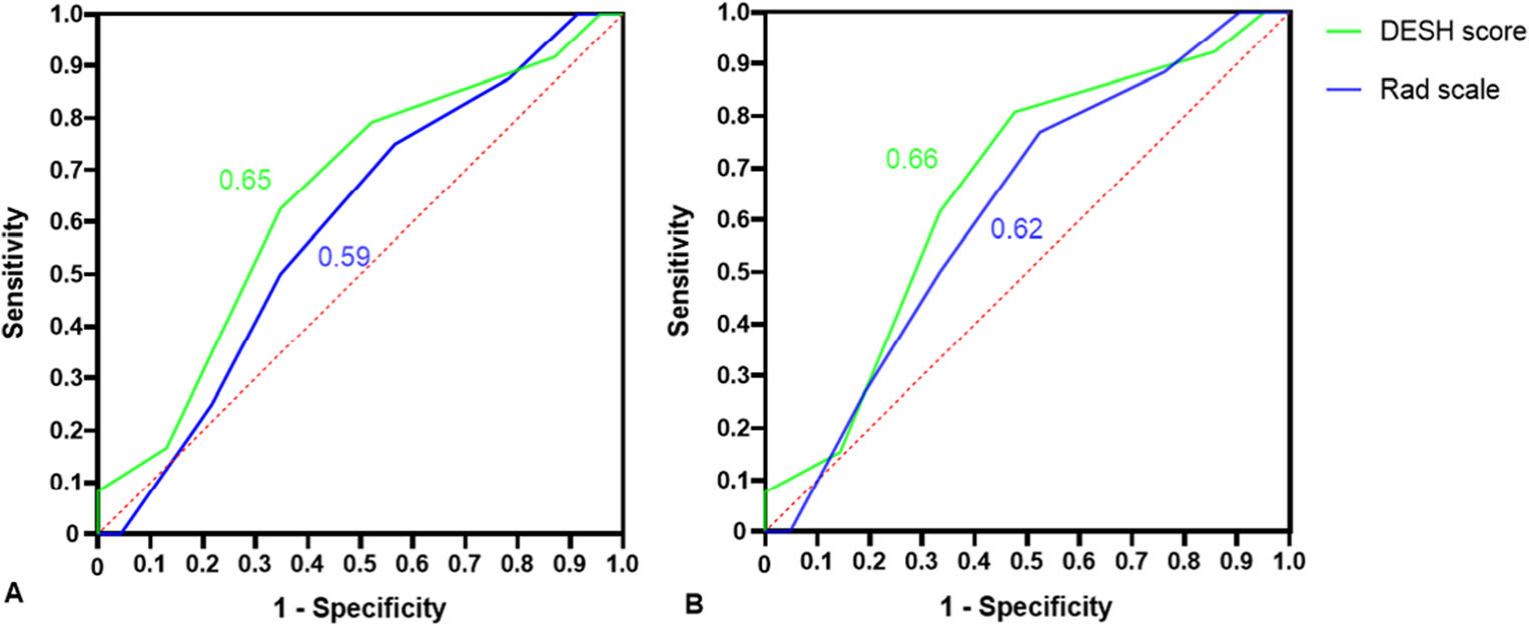
ROC and AUC of the two scores to differentiate the improvement group from the non-improvement group. **A** mRS improvement ≥ 1 as classification standard. **B** iNPHGS improvement ≥ 1 point as classification standard. ROC: receiver operating curve, AUC: area under the curve

Using different outcome indicators, the ROC analysis of the two imaging scores showed no significant difference between groups (*p* > 0.05). Under the primary result, the AUCs of the two scores were 0.65 (95% CI: 0.49−0.81, *p* = 0.079) and 0.59 (95% CI: 0.43−0.76, *p *= 0.28), respectively (Fig. 4a). Similarly, under the secondary result, the AUCs of the two scores were 0.66 (95% CI: 0.50−0.82, *p* = 0.06) and 0.62 (95% CI: 0.46−0.79, *p* = 0.15), respectively (Fig. 4b). In conclusion, the diagnostic performance for the treatment response of the two scores was poor and not significant, further corroborating our previous results.

**Fig. 5:**
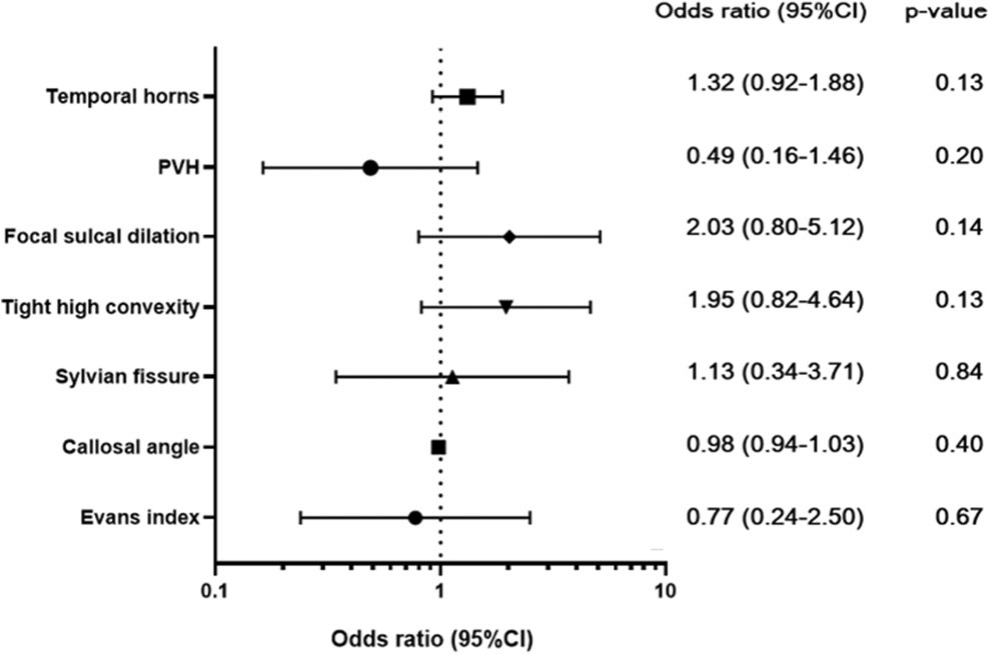
Forest plot with sex- and age-adjusted odds ratios for all imaging features. ORs with a 95% CI of 1-SD increase for continuous variables and a 1-U increase for dichotomous and ordinal variables are shown. The Sylvian fissure, narrow sulci, focally enlarged sulci, and PVH ordinal is the ordinal scale 0–2. PVH, periventricular hyperintensities

Binary logistic regression analysis was performed on all imaging indexes included in the two scores with the primary result as classification standard. The predicted values are expressed after adjustment for sex and age (Fig. 5). No MRI marker was significantly associated with postoperative improvement.

Furthermore, we analyzed the relationship between the preoperative imaging parameters and the severity of clinical symptoms in patients with iNPH (Supplementary Materials). The total DESH and iNPH Radscale scores were not associated with clinical symptoms. However, there were significant, but not strong, correlations between EI (*p* = 0.017) and the temporal horns diameter (*p* = 0.016) and clinical symptoms of gait disorders.

## Discussion

In this study of 47 patients with iNPH, we used quantitative imaging features (DESH score, iNPH Radscale, EI, and CA) to predict the prognosis of shunt surgery. We found that neither the DESH score nor iNPH Radscale was predictive of the postoperative outcome; high EI value, low corpus callosum angle, or high DESH score and iNPH Radscale score were not associated with a favorable outcome.

Obviously, both the DESH score and iNPH Radscale were based on the DESH sign, which is characterized by tight high-convexity and medial subarachnoid spaces and enlarged Sylvian fissures with ventriculomegaly [6]. For both scoring criteria, higher scores were associated with more unfavorable imaging findings. The iNPH Radscale has two more sub-items than the DESH score, which are the temporal angle and PVH, and the same item has different scoring standards. This is related to the differences in inclusion criteria and research purposes.

In a study by Shinoda et al, there was a significant difference between the DESH score based on MRI in patients with and without postoperative improvement, and a strong correlation between the DESH score and the degree of postoperative improvement [8]. This contradicts the results of the present study. The explanation for this difference is as follows: the DESH score is the result of quantifying some imaging features of iNPH, but some parameters are based on the subjective impressions of the scorer, such as the Sylvian fissure enlargement, the high-convex tightness, and the local expansion of the sulcus [12]. This may lead to differences in scores by raters, accounting for the difference in the research results. Furthermore, recent studies have questioned the prognostic prediction for surgery based on structural imaging [10, 11], mostly due to the uncertainty of the measurement standard for imaging markers. Ryska et al reported that the measurements of EI, CA, and other imaging markers will deviate with different angles or scanning sequence parameters, and this deviation often occurs in different centers. Therefore, a more standard and unified definition of the imaging plane or development of a computer-assisted system is warranted in the future [24]. In addition, a more important reason may be the sample bias caused by the different etiology of NPH in different regions of the world.

Kockum et al proposed the iNPH Radscale and confirmed its association with the severity of clinical symptoms in patients with iNPH [9]. However, our results showed no significant correlation between the total score of the iNPH Radscale and the clinical symptoms of iNPH patients; only gait disorder was associated with EI and temporal horns diameter, but the correlation was low. This is consistent with the report by Agerskov et al [11]. A possible explanation is that the changes in nerve function and structure are not synchronous in iNPH patients; Kockum et al only screened suspected iNPH patients using a questionnaire survey and the evaluation of clinical symptoms, but this was not confirmed by surgery. There may be other patients with similar symptoms in the sample, such as those with Alzheimer’s disease and PD. Our cases were confirmed after shunting. Moreover, the results of a recent study showed similar total scores of the iNPH Radscale for CSF tap test responders and non-responders. Therefore, iNPH Radscale does not predict clinical improvement after the CSF tap test [25]. This is consistent with the present results.

In the study of Hong et al, the DESH sign was considered a relevant factor for good results, while white matter hyperintensities, CA, and EI were not [26]. However, the iNPH sub-population with typical imaging features is only one subgroup. The rate of detection of DESH is only approximately 30–50%, and its ability to diagnose treatment response is poor [12, 16, 27]. This can lead to selection bias and exclude iNPH patients with atypical imaging findings from the operation schedule. In this case, it is unfair to discuss the prognosis of the surgery.

Recent studies have shown that the improvement of symptoms after shunt surgery is related to mild symptoms and short disease course [28], while delayed shunt surgery is related to poor improvement of symptoms [29]. Meanwhile, Wu et al compared systematic volumetric analysis with traditional structural imaging and pointed out that systematic volumetric analysis has a high classification performance for predicting the results of shunt surgery [30]. Furthermore, traditional structural imaging has been reported to have poor diagnostic performance for treatment response [10, 11, 28, 31], which is consistent with our results.

This study has some limitations. It was conducted at a single center using a single MRI scanner. However, this prevented any discrepancies related to the use of different scanners. Due to the strict differential diagnosis and robust inclusion criteria, the cohort was relatively small. Further multicenter large-sample studies are needed to verify the neuroimaging features predictive of the outcome of iNPH surgery. Furthermore, after 1-year follow-up, many patients could not return to the hospital due to the long distance, physical condition, or COVID pandemic. Thus, telephone interviews were conducted to ask patients and their families about the patient’s status to try and objectively evaluate the clinical symptoms.

## Conclusion

In our study, there were no significant differences in DESH and iNPH scale scores between iNPH patients with and without improved clinical symptoms a year after shunt surgery. Therefore, some patients with lower DESH and iNPH Radscale scores may also improve after surgery. In summary, structural imaging appears to have limited value and should, therefore, not be used to exclude patients from shunt surgery at this time.

Relationship between preoperative imaging parameters and clinical outcomes of iNPH patients

## Supplementary information

ESM 1 (DOCX 19 kb)

## Abbreviations

CA: Callosal angle
CSF: Cerebrospinal fluid
DESH: Disproportionately enlarged subarachnoid space hydrocephalus
DWMH: Deep white matter hyperintensities
EI: Evans’ index
iNPH: Idiopathic normal pressure hydrocephalus
iNPHGS: iNPH grading scale
mRS: Modified Rankin Scale
PACS: Picture archiving and communication system
PD: Parkinson’s disease
PVH: Periventricular hyperintensities

## Declarations

### Guarantor

The scientific guarantor of this publication is Jun Xia.

### Statistics and biometry

No complex statistical methods were necessary for this paper.

### Methodology

• prospective

• diagnostic or prognostic study

• performed at one institution
