# Supplementary material for: Value of MRI-based semi-quantitative structural neuroimaging in predicting the prognosis of patients with idiopathic normal pressure hydrocephalus after shunt surgery
Source: Eur Radiol. 2022 Apr 30;32(11):7800–10. doi: 10.1007/s00330-022-08733-3 (PMC9668801; doi:10.1007/s00330-022-08733-3)
Supplement: Supplementary file 1 — (DOCX 19 kb) [file 330_2022_8733_MOESM1_ESM.docx]

**Supplementary materials** Relationship between preoperative imaging parameters and clinical outcomes in iNPH patients.

| Neuroimaging Findings | iNPHGS | | | | |
| --- | --- | --- | --- | --- | --- |
|  | mRS | Total | Gait | Cognitive | Urinary |
| DESH score | 0.351 (0.085) | 0.270 (0.191) | 0.361 (0.076) | 0.223 (0.076) | 0.154 (0.461) |
| EI | 0.159 (0.448) | 0.246 (0.236) | 0.471 (0.017)* | 0.084 (0.690) | 0.100 (0.635) |
| CA | -0.70 (0.738) | -0.016 (0.941) | -0.011 (0.958) | -0.084 (0.689) | -0.032 (0.878) |
| DWMH | 0.06 (0.776) | -0.44 (0.833) | -0.351 (0.085) | 0.027 (0.899) | 0.154 (0.462) |
| PVH | 0.112 (0.594) | 0.042 (0.843) | -0.215 (0.302) | 0.088 (0.676) | 0.234 (0.261) |
| iNPH Radscale | 0.124 (0.556) | 0.127 (0.546) | 0.069 (0.743) | 0.046 (0.825) | 0.178 (0.394) |
| Temporal horns | 0.015 (0.944) | 0.174 (0.406) | 0.478 (0.016)* | -0.053 (0.802) | 0.055 (0.796) |

iNPHGS, idiopathic normal pressure hydrocephalus grading scale. mRS, modified Rankin Scale; * A significant relationship according to the Spearman correlation coefficient. EI, Evan’s index; CA, Callosal angle. DWMH, deep white matter hyperintensities. PVH, periventricular hyperintensities.
